# In vitro assessment of physiological traits and ROS detoxification pathways involved in tolerance of Damask rose genotypes under salt stress

**DOI:** 10.1038/s41598-023-45041-2

**Published:** 2023-10-18

**Authors:** Sahar Azizi, Hanifeh Seyed Hajizadeh, Ahmad Aghaee, Ozkan Kaya

**Affiliations:** 1 Department of Horticulture, Faculty of Agriculture, University of Maragheh, Maragheh, 55136-553 Iran; 2 Department of Biology, Faculty of Science, University of Maragheh, Maragheh, Iran; 3 Erzincan Horticultural Research Institute, Republic of Turkey, Ministry of Agriculture and Forestry, Erzincan, 24060 Turkey; 4 Department of Plant Sciences, North Dakota State University, 58102, Fargo, ND USA

**Keywords:** Plant sciences, Environmental sciences

## Abstract

*Rosa damascena* is one of the most important medicinal and ornamental plants in Iran which is tolerant of salinity to some extent. However, the selection of genotypes that are more tolerant to salinity will influence on Damask cultivation in salt stress-affected regions. For this purpose, a factorial experiment in a completely randomized design with three replicates was performed under in vitro conditions on four Damask rose genotypes (Atashi, Bi-Khar, Chahar-Fasl and Kashan) at 5 concentrations of NaCl (0, 25, 50, 75, and 100 mM), and the physico-chemical traits were measured 14 and 28 days after treatment.The results showed that Atashi genotype with high levels of Chl *a,* Chl *b*, total Chl content, carotenoids, relative leaf water content, proline, total soluble protein, TPC, TFC, TAA, and the highest increase in the activity of antioxidant enzymes such as GPX, APX, CAT, SOD, and POD as well as the lowest amount of hydrogen peroxide showed a better protection mechanism against oxidative damage than the other three genotypes (Bi-Khar, Chahar-Fasl and Kashan) in the 14th and 28th days by maintaining the constructive and induced activities of antioxidant enzymes, it was shown that Bi-Khar genotype had moderate tolerance and Kashan and Chahar-Fasl genotypes had low tolerance to salinity stress. In vitro selection methods can be used effectively for salt tolerant screening of Damask rose genotypes, although the same experiment should be conducted in open filed cultures to verify the in vitro experimental results.

## Introduction

Damask rose (*Rosa damascena*) is known as an aromatic plant, and most researchers consider its origin to be Bulgaria, Iran, Turkey and India^1^. Considering the various uses of Damask rose in medicinal products, perfumery and cosmetics, it has caused a lot of attention to develop its cultivation^2^. According to the ability of Damask rose to be somewhat tolerant to environmental stresses^3^ it seems that the selection of a more resistant genotypes has a greater advantage for the development of its cultivation in the mentioned regions. Different degrees of resistance to salinity have been seen among rose^4^ and pistachio^5^ genotypes which depends on various factors, including cultivar, rootstock, irrigation and planting system. Therefore, it is of vital need to screen and choose the salt tolerance genotypes for sustainable agriculture.

In recent years, drought stress is going to be widespread through the world and subsequently salinity is un-avoidable phenomenon as a secondary stress, especially in arid and semi-arid regions. Iran is one of the countries where major abiotic stresses such as drought and salinity cause yield reduction, loss of soil fertility and impossibility of agriculture in most of its parts^6^. Salinity is the major limiting factors in the soil which effect on plant performance and its chemical constituents. The harmful effects of salinity on plant growth are categorized in four items; (1) osmotic effect, (2) specific ion effects, (3) nutritional imbalance, and (4) production of reactive oxygen species (ROS)^7^. Salinity caused to increase in the cellular antioxidant pool components such as carotenoids, ascorbate, proline, total phenol content (TPC), total flavonoid content (TFC), and total antioxidant activity (TAA) especially in the tolerant variety of *A. tricolor*, while the sensitive variety accumulated the proline much more than other variety^8^. Against negative effect of salinity on morphology and physiology of plant, increase in superoxide dismutase (SOD), catalase (CAT), glutathione reductase (GR), ascorbate peroxidase (APX), and peroxidase (POD) activity under salinity have been reported also in plants^9^. It is suggested that the increment of ascorbate and APX is the sign of tolerance in plants under stress^9^. On the other hand, decrease in carbohydrates was expected because of prevention of the *Rubisco* and phosphoenol pyruvate carboxylase and malate dehydrogenase activities in plants under salinity^10^. Attia et al.^11^ concluded that the salt resistance of *R. damascena* at 100 mM NaCl was correlated the maintenance of high water and chlorophyll contents, although lipid peroxidation was occurred by increasing malondialdehyde (MDA) and hydrogen peroxide (H_2_O_2_) contents in Damask rose at 100 mM NaCl. It is also demonstrated that treatment of *R. rubiginosa* with 150–200 mM NaCl resulted in higher extent of leaf chlorosis compared to lower concentrations (25–50 mM), and it is more tolerant to salinity- induced by NaCl than CaCl_2_^12^. Our previous study showed that the tolerance threshold of Damask rose ʻKashanʼ to salinity was 50 mM (about 6.5 dS m^−1^), although the antioxidant capacity of explants exposed to 100 mM NaCl was higher than 50 mM^13^. According to the our previous studies^14–16^ the Damask rose genotypes enriched with antioxidants were screened across to drought stress. Due to the high economic value of the Damask rose as a suitable medicinal plant in dry regions^16^, it is necessary to evaluate some developmental and performance under salinity. In addition, one of the ways to select the genotypes with the best performance under abiotic stresses is to use of the tissue culture, which requires very little space and time^17^. Considering that Iran as the main origin of Damask rose, it is located in the arid belt of the world and had the ancient history of growing the mentioned medicinal and valuable ornamental plant in saline areas, it shows that Damask rose can be an effective option for the development of cultivation in saline areas.

Therefore, the aim of the present study was to investigate the effect of salinity on some physiological and biochemical traits, under in vitro culture after 14th and 28th days salinity treatment to better elucidate non-enzymatic and enzymatic antioxidative defense pathways, regarding salt-tolerant by comparing four genotypes of *R. damascena*.

## Results

### Physiological traits

**Figure 1:**
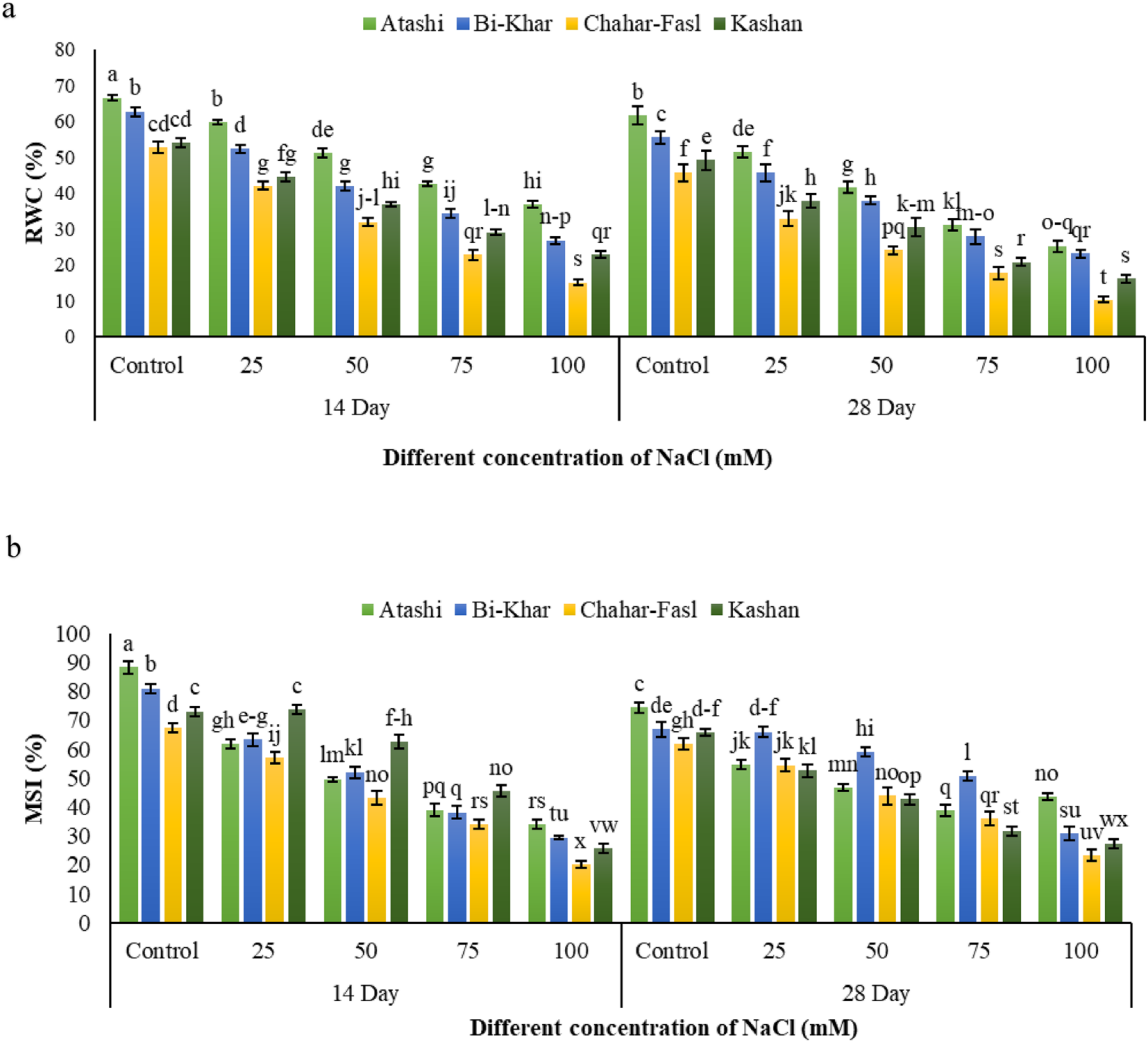
The effect of sampling time (14th and 28th day) and salinity on (**a**) RWC and (**b**) MSI of four Damask rose genotypes (Atashi, Bi-Khar, Chahar-Fasl, and Kashan). Different letters indicate significant differences according to LSD test at P < 0.05.

Salt stress in all Damask rose genotypes caused to a gradual reduction in leaf relative water content (RWC) and membrane stability index (MSI) in a concentration and time-dependent manner up to 100 mM NaCl (Fig. 1a,b) RWC decreased significantly (p < 0.05) in four genotypes under the different salt concentrations (Fig. 1a). At the 14th day, the reduction percentage in RWC in ‘Atashi’ was less (180%) at 100 mM NaCl, while ‘Chahar-Fasl’ showed a greater reduction (348%). Significant differences were observed between the 14th and 28th days, ‘Atashi’ had 244% loss and ‘Chahar-Fasl’ had 434% loss in the shoots water content (Fig. 1a). The membrane stability index (MSI) was significantly reduced in the Damask rose genotypes due to salinity stress (Fig. 1b). ‘Chahr-Fasl’ genotype had the maximum decrease (334 and 264%) while the ‘Atashi’ genotype had the minimum decrease (260 and 170%) compared to the control shoots (Fig. 1b) and other genotypes on the 14th and 28th days, respectively.

### Photosynthetic pigments

**Figure 2:**
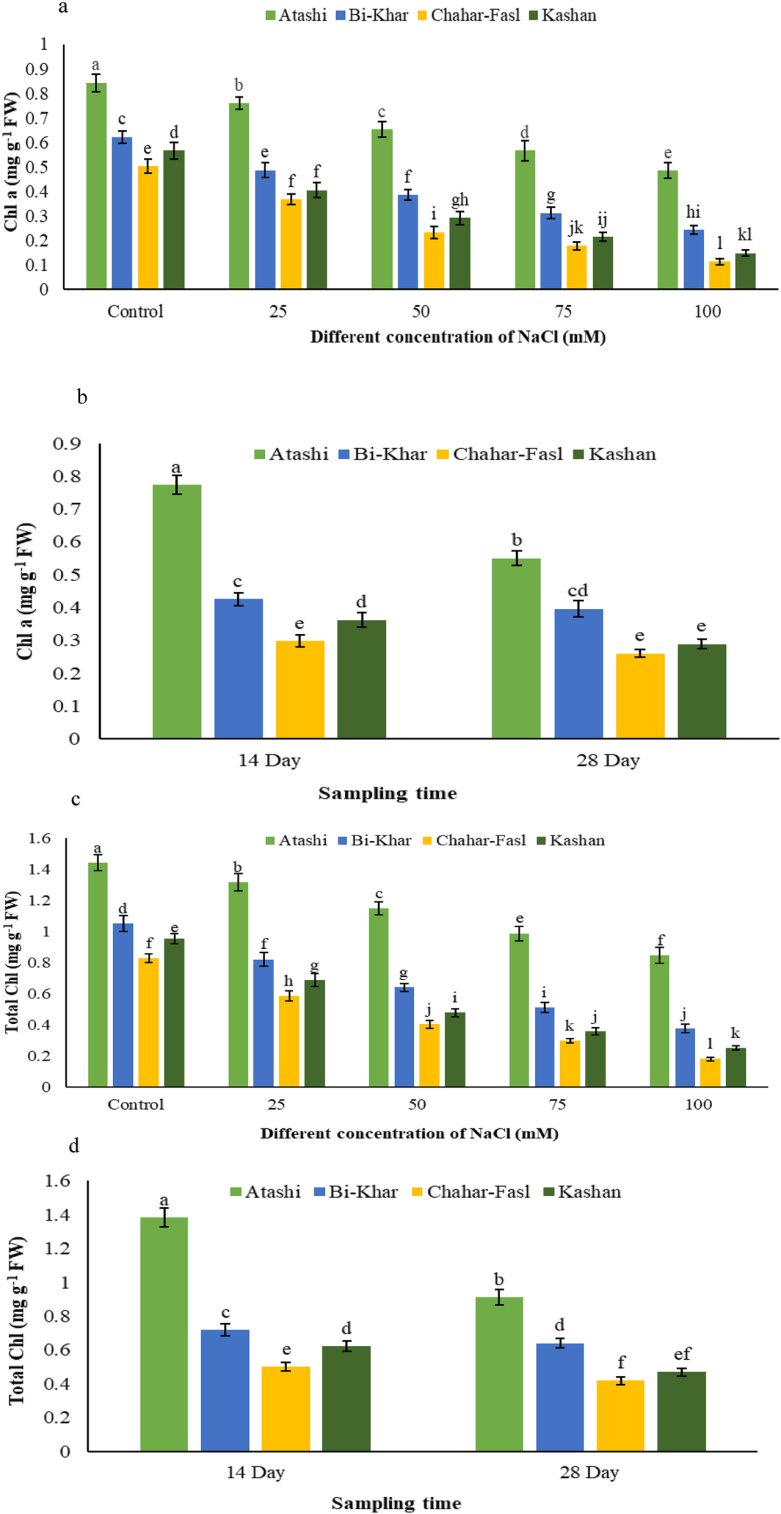
The effect of salt stress × four Damask genotypes on (**a**) Chl *a*, (**b**) sampling time (14th and 28th day) × four Damask genotypes (Atashi, Bi-Khar, Chahar-Fasl, and Kashan) on Chl *a*, (**c**) salt stress × four Damask genotypes on Chl *b*, and (**d**) sampling time × four Damask genotypes on Chl *b*. Different letters indicate significant differences according to LSD test at P < 0.05.

As well as increase in the NaCl concentration, the photosynthetic pigments content including chlorophyll *a* (Chl *a*), chlorophyll *b* (Chl *b*), total chlorophyll (total Chl,), and carotenoids (CARs) in the shoots of the plant decreased, which is an indicator of stress. The least and the highest decrease in Chl *a* were related to the ‘Atashi’ and ‘Chahar-Fasal’ with a 170% and 340%, respectively (Fig. 2a) compared to the control explants (Fig. 2a). In the 14th and 28th days, the highest Chl *a* was observed in the ‘Atashi’ and the lowest amount in both times was obtained in the ‘Chahar-Fasal’ and ‘Kashan’ (Fig. 2b). ‘Bi-Khar’ had the lowest decrease up to 7% compared to the other genotypes on the 14th day (Fig. 2b). On the other hand, ‘Chahar-Fasl’ displayed the lowest Chl *b* on the 28th day, at sever salinity stress (100 mM NaCl) compared to the control. The highest amount of total Chl (1.441 mg g^−1^ FW) was related to the ‘Atashi’ in the control shoots while the lowest amount (0.179 mg g^−1^ FW) was related to the 100 mM NaCl and ‘Chahar-Faslʼ (Fig. 2c). In comparison with the control shoots, the ‘Atashi’ and ‘Chahar-Fasl’ had the least and maximum decrease by 170% and 460%, respectively. (Fig. 2c). In the 14th and 28th days, the ‘Chahar-Fasl’ had the least total Chl (0.499 and 0.417 mg g^−1^ FW) and the ‘Atashi’ had the most total Chl (1.383 and 0.912 mg g^−1^ FW) (Fig. 2d). According to the Table 1 ‘Atashi’ displayed the least decrease, with a reduction of 150% (Table 1) in Chl *b* compared to the control on 14th day. ‘Atashi’ had the most CARs content also on 14th day under control condition, while ‘Chahar-Fasl’ and ‘Kashan’ at 100 mM NaCl on the 28th day, had the lowest CARs content. The greatest reduction in CARs were related to the ‘Chahar-Fasl’ (411%) and ‘Kashan’ (586%) on the 14th and 28th days (Table 1).

**Table 1:** Effect of NaCl and sampling time on Chl *b* and carotenoid content of Damask rose genotypes.

Time	NaCl (mM)	(14 Day)	(28 Day)	(14 Day)	(28 Day)
Genotype		Chl* b* (mg g^−1^ FW)	Chl* b* (mg g^−1^ FW)	CARs (mg g^−1^ FW)	CARs (mg g^−1^ FW)
Atashi	0	0.75 ± 0.03a	0.46 ± 0.025f	0.77 ± 0.04a	0.46 ± 0.02gh
Bikhar		0.46 ± 0.02f	0.39 ± 0.02h	0.62 ± 0.03c	0.25 ± 0.01o
Chaharfasl		0.37 ± 0.02i	0.27 ± 0.01m	0.58 ± 0.03d	0.35 ± 0.02l
Kashan		0.43 ± 0.018g	0.34 ± 0.015j	0.53 ± 0.028f	0.31 ± 0.01m
Atashi	25	0.66 ± 0.03b	0.44 ± 0.023g	0.65 ± 0.043b	0.42 ± 0.02i
Bikhar		0.35 ± 0.02j	0.3 ± 0.02k	0.45 ± 0.02h	0.19 ± 0.01q
Chaharfasl		0.23 ± 0.015n	0.19 ± 0.01p	0.42 ± 0.02i	0.28 ± 0.02n
Kashan		0.34 ± 0.022j	0.22 ± 0.02no	0.39 ± 0.022j	0.16 ± 0.009r
Atashi	50	0.61 ± 0.028c	0.37 ± 0.02hi	0.55 ± 0.03e	0.37 ± 0.01k
Bikhar		0.28 ± 0.013lm	0.22 ± 0.01no	0.37 ± 0.02k	0.13 ± 0.01st
Chaharfasl		0.18 ± 0.01pq	0.15 ± 0.008rs	0.31 ± 0.018m	0.23 ± 0.01p
Kashan		0.21 ± 0.01o	0.15 ± 0.009r	0.27 ± 0.013n	0.12 ± 0.014st
Atashi	75	0.54 ± 0.028d	0.29 ± 0.015kl	0.47 ± 0.02g	0.32 ± 0.02m
Bikhar		0.220 ± 0.01no	0.17 ± 0.01q	0.32 ± 0.01m	0.1 ± 0.01u
Chaharfasl		0.12 ± 0.009tu	0.11 ± 0.008v	0.27 ± 0.01n	0.12 ± 0.01tu
Kashan		0.17 ± 0.01q	0.11 ± 0.008v	0.22 ± 0.01p	0.087 ± 0.009v
Atashi	100	0.49 ± 0.025e	0.22 ± 0.012no	0.41 ± 0.02i	0.28 ± 0.01n
Bikhar		0.15 ± 0.009rs	0.11 ± 0.009uv	0.27 ± 0.01n	0.08 ± 0.009v
Chaharfasl		0.076 ± 0.007w	0.05 ± 0.004x	0.14 ± 0.009s	0.06 ± 0.007w
Kashan		0.13 ± 0.01st	0.06 ± 0.005wx	0.17 ± 0.009r	0.05 ± 0.006w
SOV					
Genotype (G)		**	**	**	**
Salinity (S)		**	**	**	**
G × S		**	**	**	**
Time (T)		**	**	**	**
G × T		**	**	**	**
S × T		**	**	**	**
G × S × T		*	*	**	**
Error		0.0001	0.0001	0.0001	0.0001
CV		6.22	6.22	4.28	4.28

Different letters indicate significant differences in each trait according to LSD test at P < 0.05. ns, * and ** indicate no significant difference, significant at 5% probability level and significant at 1% probability level, respectively. S. O. V. and CV refer to the source of variation and coefficient variation, respectively.

### Antioxidant enzyme activities

Antioxidant enzyme activities in Damask rose genotypes were affected by NaCl concentration and time (Table 2) as in the 14th day up to 100 mM NaCl and in the 28th day up to 75 mM NaCl, all of the salt-treated Damask rose genotypes showed a substantial increase in their guaiacol peroxidase (GPX) and SOD concentration (Table 2). Furthermore, ‘Bi-Kharʼ and ‘Kashanʼ at 100 mM NaCl showed the maximum (626%) and the minimum (387%) increment in GPX on the 14th day, respectively compared to the control. At 75 mM NaCl, ʻAtashiʼ and ʻKashanʼ had the greatest (258%) and the lowest (119%) increment on the 28th day (Table 2). The activity of SOD in ʻAtashiʼ and ʻBi-Kharʼ showed the greatest and lowest amount (429% and 200%, respectively) in comparison with the control shoots on the 14th day, which were followed up to 204% and 160%, respectively on the 28th day (Table 2). Polyphenol oxidase (PPO) activity in all genotypes was increased at 75 and 50 mM level of NaCl on the 14th and 28th days, although it was decreased at higher concentrations of NaCl (Table 2). The highest PPO activity was obtained in ‘Atashi’ under 50 mM NaCl on the 28th day, while the lowest activity was obtained in the control shoots on the 14th day. Finally, the highest increment was recorded in ‘Atashi’ (630%), and the lowest was recorded in ‘Kashan’ (390%) on the 28th day (Table 2).

**Table 2:** Effect of NaCl and sampling time on GPX, SOD, PPO, CAT and APX activity of Damask rose genotypes.

Time	NaCl (mM)	(14 day)	(28 day)	(14 day)	(28 day)	(14 day)	(28 day)
Genotype		GPX (unit mg^−1^ protein)	GPX (unit mg^−1^ protein)	SOD (unit mg^−1^ protein)	SOD (unit mg^−1^ protein)	PPO (unit mg^−1^ protein)	PPO (unit mg^−1^ protein)
Atashi	0	0.19 ± 0.01v–x	0.78 ± 0.04l–o	0.31 ± 0.015vw	0.92 ± 0.06h–j	0.02 ± 0.001u–w	0.076 ± 0.05g–j
Bikhar		0.15 ± 0.01wx	0.65 ± 0.04p–r	0.49 ± 0.02q–u	0.81 ± 0.05j–l	0.018 ± 0.001vw	0.026 ± 0.001u–w
Chaharfasl		0.14 ± 0.01x	0.38 ± 0.01t	0.18 ± 0.01x	0.38 ± 0.02uv	0.016 ± 0.001w	0.015 ± 0.001w
Kashan		0.23 ± 0.02u–w	0.57 ± 0.02rs	0.44 ± 0.02s–v	0.73 ± 0.04k–n	0.015 ± 0.001w	0.018 ± 0.001vw
Atashi	25	0.41 ± 0.03t	0.93 ± 0.06ij	0.4 ± 0.02t–v	1.08 ± 0.08e–g	0.033 ± 0.002r–v	0.12 ± 0.007e
Bikhar		0.29 ± 0.02u	0.85 ± 0.05j–l	0.62 ± 0.03m–q	0.95 ± 0.06g–i	0.026 ± 0.001s–w	0.039 ± 0.003o–t
Chaharfasl		0.26 ± 0.02uv	0.54 ± 0.02s	0.22 ± 0.01wx	0.45 ± 0.02r–u	0.032 ± 0.002r–u	0.027 ± 0.001s–w
Kashan		0.43 ± 0.03t	0.72 ± 0.03n–p	0.58 ± 0.025o–r	0.84 ± 0.05i–k	0.024 ± 0.001t–w	0.034 ± 0.002q–v
Atashi	50	0.70 ± 0.05n–q	1.26 ± 0.08cd	0.61 ± 0.03n–q	1.26 ± 0.08cd	0.048 ± 0.002l–r	0.48 ± 0.02a
Bikhar		0.64 ± 0.04p–r	1.08 ± 0.08fg	0.76 ± 0.04k–m	1.09 ± 0.07ef	0.043 ± 0.002n–r	0.16 ± 0.008c
Chaharfasl		0.53 ± 0.03s	0.63 ± 0.03qr	0.54 ± 0.03p–t	0.57 ± 0.03o–s	0.042 ± 0.002n–s	0.088 ± 0.005fg
Kashan		0.65 ± 0.04p–r	0.83 ± 0.05k–m	0.69 ± 0.03l–o	0.93 ± 0.05h–j	0.036 ± 0.001p–u	0.1 ± 0.006f
Atashi	75	0.85 ± 0.07j–l	2.02 ± 0.1a	0.61 ± 0.03n–q	1.89 ± 0.16a	0.099 ± 0.007fg	0.34 ± 0.02b
Bikhar		0.79 ± 0.06–n	1.61 ± 0.1b	0.87 ± 0.05i–k	1.39 ± 0.1bc	0.072 ± 0.004h–k	0.12 ± 0.007e
Chaharfasl		0.77 ± 0.05–o	0.84 ± 0.05k–m	0.65 ± 0.03m–p	0.74 ± 0.04k–n	0.08 ± 0.005gh	0.07 ± 0.004h–k
Kashan		0.75 ± 0.05m–o	1.19 ± 0.08de	0.81 ± 0.05j–l	1.21 ± 0.08de	0.061 ± 0.03j–m	0.078 ± 0.004g–i
Atashi	100	1.12 ± 0.08ef	1.66 ± 0.15b	1.34 ± 0.07b–d	1.42 ± 0.12b	0.064 ± 0.004i–l	0.19 ± 0.008d
Bikhar		0.98 ± 0.07hi	1.3 ± 0.085c	0.99 ± 0.06f–i	1.23 ± 0.1d	0.057 ± 0.003k–n	0.063 ± 0.004i–l
Chaharfasl		0.7 ± 0.05o–q	0.75 ± 0.04m–o	0.8 ± 0.05j–l	0.66 ± 0.02m–p	0.053 ± 0.003l–o	0.046 ± 0.002m–r
Kashan		0.90 ± i–k	1.03 ± 0.08gh	0.9 ± 0.065ij	1.04 ± 0.07f–h	0.05 ± 0.002l–q	0.051 ± 0.003l–p
SOV							
Genotype (G)		**	**	**	**	**	**
Salinity (S)		**	**	**	**	**	**
G × S		**	**	**	**	**	**
Time (T)		**	**	**	**	**	**
G × T		**	**	**	**	**	**
S × T		**	**	*	*	**	**
G × S × T		**	**	**	**	**	**
Error		0.003	0.003	0.007	0.007	0.0001	0.0001
CV		6.61	6.61	10.30	10.30	26	26

Time	NaCl (mM)	(14 day)	(28 day)	(14 day)	(28 day)		
Genotype		CAT (unit mg^−1^ protein)	CAT (unit mg^−1^ protein)	APX (unit mg^−1^ protein)	APX (unit mg^−1^ protein)		
Atashi	0	0.18 ± 0.01w	0.42 ± 0.02o–r	0.078 ± 0.004st	0.2 ± 0.01j		
Bikhar		0.28 ± 0.01t–v	0.33 ± 0.02st	0.055 ± 0.002vw	0.12 ± 0.007p		
Chaharfasl		0.25 ± 0.01v	0.23 ± 0.01vw	0.027 ± 0.001x	0.06 ± 0.003uv		
Kashan		0.24 ± 0.008v	0.31 ± 0.01tu	0.043 ± 0.002wx	0.096 ± 0.005qr		
Atashi	25	0.32 ± 0.02tu	0.62 ± 0.04ij	0.11 ± 0.007pq	0.24 ± 0.015g		
Bikhar		0.39 ± 0.02qr	0.45 ± 0.02op	0.081 ± 0.005r–t	0.17 ± 0.008lm		
Chaharfasl		0.29 ± 0.01tu	0.38 ± 0.02rs	0.05 ± 0.002vw	0.09 ± 0.005rs		
Kashan		0.27 ± 0.01tu	0.43 ± 0.03o–q	0.071 ± 0.003u	0.147 ± 0.007o		
Atashi	50	0.44 ± 0.02o–q	0.78 ± 0.04e–g	0.15 ± 0.008no	0.29 ± 0.02e		
Bikhar		0.53 ± 0.03lm	0.61 ± 0.03jk	0.12 ± 0.07p	0.23 ± 0.015gh		
Chaharfasl		0.4 ± 0.02p–r	0.51 ± 0.035mn	0.083 ± 0.005r–t	0.14 ± 0.007o		
Kashan		0.47 ± 0.02no	0.6 ± 0.038jk	0.1 ± 0.005pq	0.22 ± 0.008i		
Atashi	75	0.6 ± 0.04jk	1.42 ± 0.07a	0.19 ± 0.0085jk	0.96 ± 0.045a		
Bikhar		0.67 ± 0.03hi	1.12 ± 0.06b	0.15 ± 0.007no	0.44 ± 0.02c		
Chaharfasl		0.57 ± 0.03kl	0.74 ± 0.05g	0.115 ± 0.006p	0.24 ± 0.01gh		
Kashan		0.63 ± 0.04ij	0.93 ± 0.05c	0.14 ± 0.007o	0.31 ± 0.015d		
Atashi	100	0.92 ± 0.05c	1.08 ± 0.05b	0.25 ± 0.01i	0.54 ± 0.03b		
Bikhar		0.82 ± 0.04de	0.85 ± 0.05d	0.18 ± 0.08kl	0.28 ± 0.01ef		
Chaharfasl		0.65 ± 0.03h	0.63 ± 0.04ij	0.14 ± 0.06o	0.18 ± 0.0085k		
Kashan		0.75 ± 0.043fg	0.8 ± 0.042ef	0.16 ± 0.065mn	0.27 ± 0.01f		
SOV							
Genotype (G)		**	**	**	**		
Salinity (S)		**	**	**	**		
G × S		**	**	**	**		
Time (T)		**	**	**	**		
G × T		**	**	**	**		
S × T		**	**	**	**		
G × S × T		**	**	**	**		
Error		0.001	0.001	0.0001	0.0001		
CV		4.35	4.35	11.05	11.05		

Different letters indicate significant differences in each trait according to LSD test at P < 0.05. ns, * and ** indicate no significant difference, significant at 5% probability level and significant at 1% probability level, respectively. S. O. V. and CV refer to the source of variation and coefficient variation, respectively.

The activity of CAT and APX was increased in all Damask rose genotypes on 14th day and then reduced at higher concentrations of NaCl on 28th day. The most and the least CAT activity was detected in ‘Atashi’ under 75 mM NaCl on the 28th day and control treatment, respectively (Table 2). Our findings revealed that the highest increase was noted in ‘Atashi’ (420%) and the lowest was recorded in ‘Chahar-Fasl’ (250%) on 14th day (Table 2). The most APX activity was observed in ‘Atashi’ under 75 mM NaCl on 28th day, while the least was observed in ‘Kashan’ under control conditions on 14th day. Our findings showed that the highest and the lowest increase in APX were obtained in ʻAtashiʼ (470%) and ‘Kashan’ (330%) on 28th day, respectively (Table 2).

**Figure 3:**
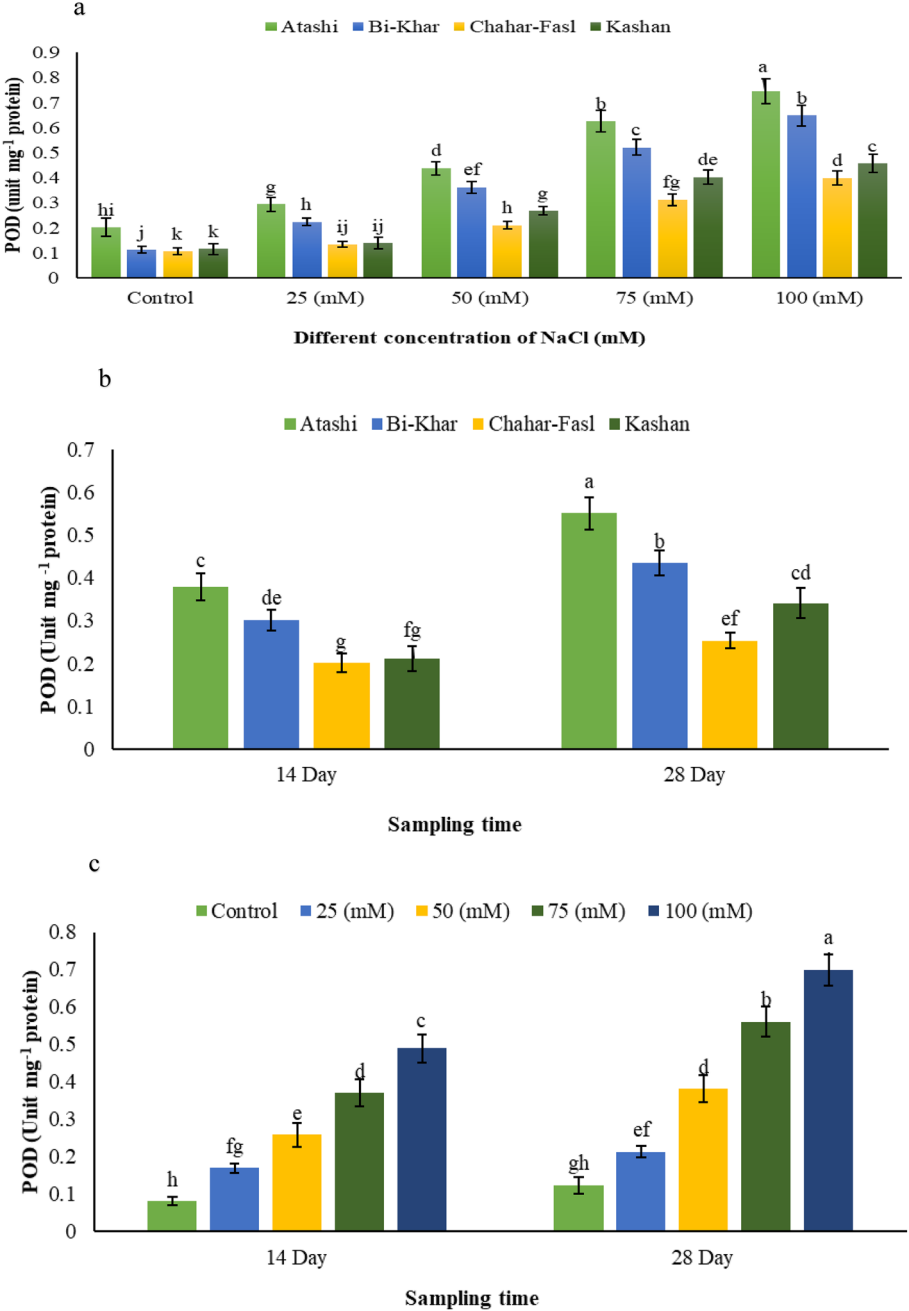
The effect of salt stress × four Damask genotypes on (**a**) POD activity, (**b**) sampling time (14th and 28th day) × four Damask genotypes (Atashi, Bi-Khar, Chahar-Fasl, and Kashan) on POD activity, and (**c**) salt stress × sampling time on POD activity. Different letters indicate significant differences according to LSD test at P < 0.05.

POD enzyme activity also increased along with increase in the level of NaCl, so that it showed the maximum and minimum value respectively in ʻAtashiʼ and ʻKashanʼ at 100 mM NaCl (Fig. 3a). ʻAtashiʼ had the most POD activity on both times after treatment, while the least POD activity was related to ʻChahar-Faslʼ. (Fig. 3b). POD showed the maximum activity 28 days after sever salinity treatment while the lowest POD activity was observed in the control shoots on 14th day (Fig. 3c).

### Total phenol content (TPC) and total flavonoid content (TFC)

The triple interaction between time, genotype, and salinity had a statistically significant (P ≤ 0.05) impact on TPC and TFC content (Table 3) of Damask rose genotypes. Along with increase in the concentration of NaCl, the level of TPC also increased in all genotypes as the highest TPC was obtained in Atashi genotype under 100mM NaCl on 28th day (Table 3). The lowest TPC content was observed in the control explants of ‘Chahar-Fasl’ and ‘Kashan’, on 14th day. A marked enhancement of TPC content (324%) belonged to ‘Atashi’ under 100 mM NaCl on 14th day and the lowest was displayed in ‘Bi-Khar’ (176%) on 28th day (Table 3). In the 14th day up to 100 mM NaCl and in the 28th day up to 75 mM salinity, all of the salt treated Damask rose genotypes showed a substantial increase in their TFC (Table 3). Also, ‘Atashi’ and ‘Kashan’ at 100 mM NaCl showed the greatest (270%) and the lowest (387%) increment in TFC on 14th day, respectively compared to the control shoots. On the other hand, under 75 mM NaCl, ‘Atashi’ and ‘Chahar-Fasl’ had the greatest (258%) and the lowest (265%) increment in TFC on 28th day (Table 3).

**Table 3:** Effect of NaCl and sampling time (14th and 28th day) on total phenol content (TPC), total flavonoid content (TFC) and total antioxidant activity (TAA) of Damask rose genotypes.

Time	NaCl (mM)	(14 day)	(28 day)	(14 day)	(28 day)	(14 day)	(28 day)
Genotype		TPC (mg GAE g^−1^ FW)	TPC (mg GAE g^−1^ FW)	TFC (mg QE g^−1^ FW)	TFC (mg QE g^−1^ FW)	TAA (%)	TAA (%)
Atashi	0	7.16 ± 0.55uv	9.7 ± 0.7rs	2.32 ± 0.3u	4.42 ± 0.36l	24.31 ± 1.3uv	42.93 ± 2.3mn
Bikhar		6.02 ± 0.4vw	14.92 ± 0.8j	2.05 ± 0.2v	3.53 ± 0.3p	28.45 ± 1.6st	39.31 ± 1.5p
Chaharfasl		4.32 ± 0.3x	11.16 ± 0.65o–r	1.8 ± 0.1w	2.03 ± 0.13v	19.92 ± 1.03w	26.17 ± 1.2tu
Kashan		4.96 ± 0.35wx	12.9l ± 0.8–n	1.47 ± 0.1x	3.18 ± 0.2qr	20.15 ± 1.3w	35.19 ± 1.9q
Atashi	25	9.97 ± 0.64s	13.23 ± 0.85k–m	3.22 ± 0.35q	5.56 ± 0.4gh	40.08 ± 1.8op	51.43 ± 2.5h–j
Bikhar		7.85 ± 0.45tu	17.84 ± 0.95gh	2.93 ± 0.22s	4.59 ± 0.37k	34.59 ± 1.5q	44.9 ± 2lm
Chaharfasl		6.27 ± 0.36v	14.72 ± 0.9j	2.62 ± 0.3t	2.38 ± 0.18u	23.43 ± 1.3v	30.94 ± 1.5rs
Kashan		6.57 ± 0.42v	16.87 ± 0.82hi	2.03 ± 0.15v	4.07 ± 0.26m	27.82 ± 1.7t	41.18 ± 2.1n–p
Atashi	50	12.21 ± 0.8m–o	16.44 ± 0.96i	4.91 ± 0.4i	6.18 ± 0.45e	48.93 ± 2.3jk	58.47 ± 2.1ef
Bikhar		9.87 ± 0.5s	21.6 ± 1.5e	3.84 ± 0.3no	5.63 ± 0.34g	45.54 ± 1.8l	55.06 ± 1.7g
Chaharfasl		8.38t ± 0.48	18.31 ± 1.1g	3.29 ± 0.28q	3.76 ± 0.26no	31.38 ± 1.6r	36.68 ± 2.3q
Kashan		8.52t ± 0.5	19.57 ± 1.3f	2.65 ± 0.2t	4.65 ± 0.32jk	36.49 ± 1.9q	51.61 ± 2.5hi
Atashi	75	14.13 ± 0.7jk	19.59 ± 1.2f	5.44 ± 0.45h	11.74 ± 0.6a	54.48 ± 2.6g	63.75 ± 2.6cd
Bikhar		11.27 ± 0.6o–q	24.9 ± 1.9c	4.73 ± 0.41jk	7.78 ± 0.5b	53.96 ± 2.3gh	61.44 ± 2.2cd
Chaharfasl		10.6 ± 0.55p–s	23.31 ± 1.3d	3.71 ± 0.3o	4.77 ± 0.38ij	39.96 ± 2.1op	49.12 ± 2.06i–k
Kashan		12.12 ± 0.7q–s	24.91 ± 1.8c	3.07 ± 0.3rs	6.3 ± 0.42e	43.65 ± 2.2l–n	64.58 ± 2.6c
Atashi	100	23.25 ± 1.2d	28.74 ± 2.1a	6.27 ± 0.5e	6.7 ± 0.5c	67.42 ± 2.2b	84.26 ± 2.8a
Bikhar		14.81 ± 0.7j	26.34 ± 1.6b	5.81 ± 0.4f	6.55 ± 0.43d	60.9 ± 1.5de	67.63 ± 1.9b
Chaharfasl		13.43 ± 0.63kl	21.71 ± 1.5e	4.28l ± 0.43	4.34 ± 0.32l	48.68 ± 1.8k	42.16 ± 1.7no
Kashan		11.75 ± 0.75n–p	23.29 ± 1.6d	3.86 ± 0.34n	5.87 ± 0.36f	52.8 ± 2gh	58.03 ± 2.4f
SOV							
Genotype (G)		**	**	**	**	**	**
Salinity (S)		**	**	**	**	**	**
G × S		**	**	**	**	**	**
Time (T)		**	**	**	**	**	**
G × T		**	**	**	**	**	**
S × T		**	**	*	*	**	**
G × S × T		**	**	*	*	*	*
Error		0.528	0.528	0.008	0.008	2.519	2.519
CV (%)		5.05	5.05	4.11	4.11	6.56	6.56

Different letters indicate significant differences in each trait according to LSD test at P < 0.05. ns, * and ** indicate no significant difference, significant at 5% probability level and significant at 1% probability level, respectively. S. O. V. and CV refer to the source of variation and coefficient variation, respectively.

### Total antioxidant activity (TAA)

 TAA was significantly affected by time, genotype and salt stress (Table 3). In all four genotypes, an enhancement in TAA was observed by increasing in NaCl concentration compared to the control. The highest TAA was obtained at 100 mM NaCl of the ‘Atashi’ on 28th day and the lowest was recorded for ‘Chahar-Fasl’ and ‘Kashan’ control shoots on 14th day. The most significant increase in TAA was obtained in ‘Atashi’ (277%) under the highest concentration of NaCl on 14th day (Table 3).

### *Protein, proline, malondialdehyde (MDA) and hydrogen peroxide (H*_*2*_*O*_*2*_*) content*

The mean comparison for the interaction and main effects of salinity, time and genotype on protein, proline, H_2_O_2_, and MDA is presented in Table 4. Up to 25 mM NaCl, the amount of total soluble protein increased in all genotypes except for ‘Atashi’. As well as increase in the NaCl concentration, there was a decreasing trend in the amount of total soluble protein in all Damask rose genotypes (Table 4). ‘Atashiʼ had the minimum decrease in total protein up to 39.7% and 60.6% on 14th and 28th days, respectively, while ‘Chahar-Fasl had the maximum decrease with 230% and 340% on 14th and 28th days, respectively, compared to the other genotypes (Table 4). On 14th and 28th days of the experiment, increasing in the NaCl concentration triggered a substantial increase in the amount of proline in all Damask rose genotypes. ‘Atashi’ and ‘Bi-Khar’ had the maximum and minimum proline at 100 mM NaCl, compared to the control on 28th and 14th days, respectively (Table 4). As well as increase in the concentration of NaCl from 0 to 100 mM, the amount of H_2_O_2_ increased dramatically in all Damask rose genotypes. At 100 mM NaCl, ‘Kashan’ showed the maximum H_2_O_2_ with 236% and 264% compared to the control conditions on 14th and 28th days, respectively while ‘Atashi’ showed the minimum increase up to 212% compared to the control Damask explants (Table 4). All of the salt-treated Damask rose genotypes showed a significant increment in their MDA content on 14th and 28th days (Table 4) as ‘Bi-Khar’ and ‘Atashi’ showed the most (380%) and the least (230%) increment at 100 mM NaCl compared to the normal condition (no salinity) on 28th and 14th days, respectively (Table 4).

**Table 4:** Effect of NaCl and sampling time on protein, proline, H_2_O_2_ and MDA of Damask rose genotypes.

Time	NaCl (mM)	(14 day)	(28 day)	(14 day)	(28 day)	(14 day)	(28 day)	(14 day)	(28 day)
Genotype		Protein (mg g^−1^ FW)	Protein (mg g^−1^ FW)	Proline (μmol g^−1^ FW)	Proline (μmol g^−1^ FW)	H_2_O_2_ (μg L^−1^)	H_2_O_2_ (μg L^−1^)	MDA (μmol g^−1^ FW)	MDA (μmol g^−1^ FW)
Atashi	0	2.12 ± 0.2c	1.58 ± 0.1i	15.37 ± 1.2k	12.63 ± 1.1n–p	1.69 ± 0.2u	1.91 ± 0.1st	1.18 ± 0.1wx	1.33 ± 0.12x
Bikhar		1.87 ± 0.1f	1.42 ± 0.1k	12.53 ± 1.1n–p	9.54 ± 0.85rs	1.81 ± 0.25tu	1.72 ± 0.1u	1.23 ± 0.1w	1.005 ± 0.07y
Chaharfasl		1.49 ± 0.1j	0.91 ± 0.07r	7.31 ± 0.85tu	3.39 ± 0.4x	2.09 ± 0.23qr	2.11 ± 0.15qr	2.24 ± 0.151r	2.06 ± 0.13s
Kashan		1.84 ± 0.2fg	1.08 ± 0.08p	4.39 ± 0.6wx	7.53 ± 0.6tu	1.83 ± 0.17tu	1.88 ± 0.12tu	1.64 ± 0.12uv	3.63 ± 0.23k
Atashi	25	2.35 ± 0.3b	1.82 ± 0.16g	21.2 ± 1.5i	18.14 ± 1.3j	2 ± 0.15 r–t	2.2 ± 0.16q	1.38 ± 0.1w	1.74 ± 0.1tu
Bikhar		2.37 ± 0.2b	1.62 ± 0.12i	14.4 ± 1.2k–m	14.54 ± 1.1k–m	1.95 ± 0.2r–t	2.07 ± 0.13q–s	1.75 ± 0.11tu	1.54 ± 0.08v
Chaharfasl		1.76 ± 0.13h	1.22 ± 0.1o	9.136 ± 0.8rs	5.05 ± 0.5vw	2.74 ± no	2.52 ± 0.17p	2.89 ± 0.23n	2.73 ± 0.24op
Kashan		2.07 ± 0.15d	1.32 ± 0.09mn	6.06 ± 0.55uv	8.36 ± 0.7st	2.442p	2.25 ± 0.18q	2.34 ± 0.14qr	5.33 ± 0.4d
Atashi	50	2.52 ± 0.23a	1.39 ± 0.1kl	24.15 ± g1.3h	22.79 ± 1.5h	2.6 ± 0.27op	2.58 ± 0.2op	1.82 ± 0.09t	2.66 ± 0.2p
Bikhar		1.52 ± 0.12j	1.23 ± 0.09o	18.15 ± 1.6j	19.96 ± 1.25i	2.87 ± 0.28mn	2.85 ± 0.22mn	2.46 ± 0. 25qr	2.48 ± 0.21q
Chaharfasl		1.26 ± 0.1o	0.63 ± 0.05t	11.61 ± 1.1pq	7.312 ± 0.62tu	3.68 ± 0.3hi	3.64 ± 0.28hi	3.36 ± 0.28kl	3.27 ± 0.23lm
Kashan		1.52 ± 0.16j	0.81 ± 0.07s	8.45 ± 0.9st	14.75 ± 1.1kl	3.3 ± 0.2k	3.08 ± 0.27k	2.87 ± 0.18no	5.92 ± 0.35c
Atashi	75	1.98 ± 0.14e	1.23 ± 0.09o	27.84 ± 1.8de	28.24 ± 1.6d	3.1 ± 0.2l	3.53 ± 0.23l	2.43 ± 0.2qr	3.2 ± 0.2m
Bikhar		1.27 ± 0.0.1no	1.1 ± 0.08p	20.66 ± 1.33i	25.84 ± 1.32f	3.35 ± 0.24jk	3.9 ± 0.3jk	2.94 ± 0.22n	3.27 ± 0.16lm
Chaharfasl		1.08 ± 0.08pq	0.48 ± 0.03u	13.72 ± 1.13l–n	9.89 ± 0.6r	4.12 ± 0.32e	4.72 ± 0.32e	4.08 ± 0.3g	4.68 ± 0.23e
Kashan		1.35 ± 0.09lm	0.63 ± 0.05t	10.31 ± 0.95qr	20.9 ± 1.1i	3.86 ± 0.25gh	4.08 ± 0.25gh	3.47 ± 0.02jk	6.86 ± 0.34b
Atashi	100	1.81 ± 0.1gh	1.13 ± 0.09p	43.66 ± 1.8b	47.31 ± 1.7a	3.59 ± 0.23i	4.06 ± 0.3i	2.8 ± 0.16n	3.44 ± 0.21i
Bikhar		1.03 ± 0.095q	0.93 ± 0.07r	25.42 ± 1.3fg	32.77 ± 1.38c	3.92 ± 0.21fg	4.42 ± 0.33fg	3.51 ± 0.22ij	3.82 ± 0.28h
Chaharfasl		0.93 ± 0.07r	0.35 ± 0.03v	17.93 ± 1.1j	11.96 ± 0.9op	4.74 ± 0.35c	5.48 ± 0.4c	4.87 ± 0.32f	6.02 ± 0.361c
Kashan		1.1 ± 0.08p	0.5 ± 0.04u	12.26 ± 1.1m–o	25.7 ± 1.2f	4.34 ± 0.25d	4.97 ± 0.35d	3.9 ± 0.22h	9.53 ± 0.45a
SOV									
Genotype (G)		**	**	**	**	**	**	**	**
Salinity (S)		**	**	**	**	**	**	**	**
G × S		**	**	**	**	**	**	**	**
Time (T)		**	**	**	**	**	**	**	**
G × T		**	**	**	**	**	**	**	**
S × T		**	**	**	**	**	**	**	**
G × S × T		**	**	**	**	**	**	**	**
Error		0.001	0.001	0.728	0.728	0.013	0.013	0.008	0.008
CV		6.71	6.71	5.23	5.23	3.74	3.74	7.76	7.76

Different letters indicate significant differences in each trait according to LSD test at P < 0.05. ns, * and ** indicate no significant difference, significant at 5% probability level and significant at 1% probability level, respectively. S. O. V. and CV refer to the source of variation and coefficient variation, respectively.

### Multivariate analysis of Damask rose genotypes under NaCl treatment and sampling time

**Figure 4:**
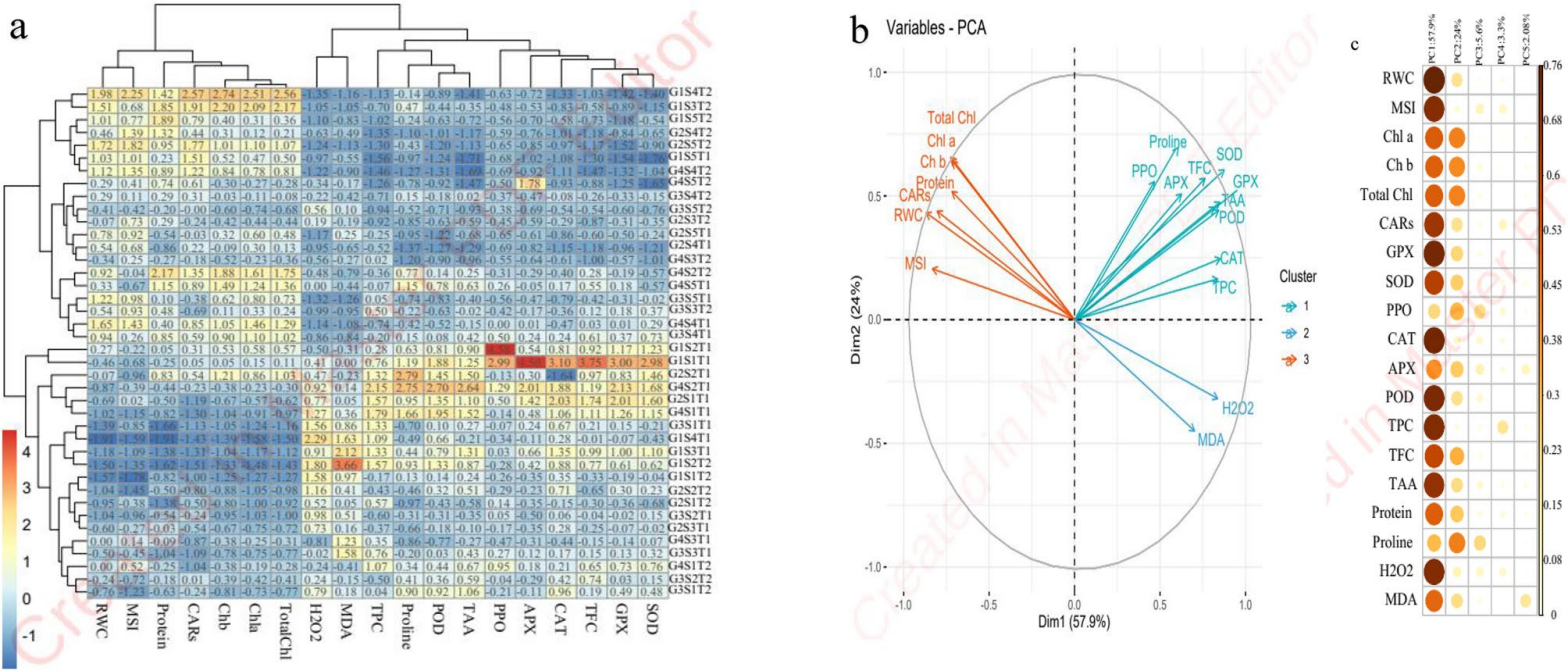
Heat map (**a**), loading biplot of the evaluated traits (**b**) and principal component analysis heat map (**c**) of the enzymatic antioxidants, the physiological and the biochemical changes in Damask rose genotypes. Genotypes under in vitro-induced salt stress condition. Heat map representing of relative water content (RWC), membrane stability index (MSI), Chlorophyll *a* (Chl *a*), Chlorophyll *b* (Chl *b*), Total chlorophyll (Total Chl), Carotenoids (CARs), Proline, Malondialdehyde (MDA), hydrogen peroxide (H_2_O_2_), Total soluble protein, Guaiacol peroxidase (GPX), Ascorbate peroxidase (APX), Superoxide dismutase (SOD), Peroxidase (POD), Catalase (CAT), Polyphenol oxidase (PPO), Total flavonoids content (TFC), Total phenol content (TPC) and Total antioxidant activity (TAA).

Heat map analysis based on the response of Damask rose genotypes to salt stress simulated by NaCl applications under in vitro condition revealed that the traits including MDA, H_2_O_2_, proline, TAA, and TPC had positive correlation to salt stress tolerance. On the other hand, some traits such as enzymatic antioxidant including POD, SOD, CAT, APX, and PPO along with TFC, photosynthetic pigment, protein, RWC and MSI were also correlated positively (Fig. 4a). Cluster analysis and dendrograms in the heat map (Fig. 4b) revealed three major classes in the assessed traits of the plants under salt stress. Class I contained PPO, Proline, APX, TFC, SOD, GPX, TAA, POD CAT and TPC; class II contained H_2_O_2_ and MDA and class III contained MSI, RWC, CARs, Protein, Chl *a*, Chl *b* and Total Chl (Fig. 4b). Moreover, biplot of variables confirmed the heat map cluster analysis in which traits classified in three groups (Fig. 4a). In general, cluster analysis of heat maps for interaction between NaCl and sampling time indicated three classes. Class I contained the Damask rose genotypes was treated by NaCl and 14th day after treatment. Class II contained the Damask rose genotypes was treated by NaCl and 28th day. Finally, class III *R. damascena* genotypes under severe salt stress which was induced by 100 mM NaCl (Fig. 4a).

The principal component analysis (PCA) and the proportion of total variance (Fig. 4c) explained that three PCA were contributing 87.5% of total variation. The first PCA was the most influential with the variance of 57.9% and contained the traits of CARs, TPD, PPO, Proline, SOD, CAT, APX, GPX and POD activity. The second PCA explained 24% of the total variance, and consisted of RWC, MSI, CARs, Protein Chl *a*, Chl *b* and total Chl. The third PCA explained 5.6% of the total variance with H_2_O_2_ and MDA (Fig. 4c).

**Figure 5:**
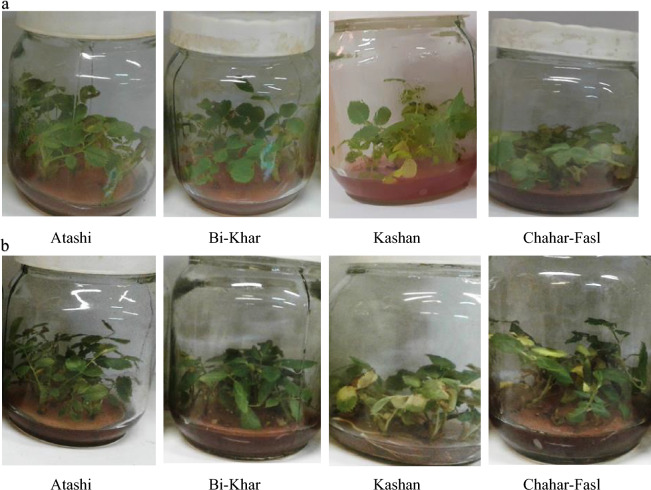
Comparison of Damask rose genotypes (Atashi, Bi-Khar, Chahar-Fasl, and Kashan) under 100 mM salinity on the 14th day (**a**) and 28th day (**b**) after salinity treatment.

Difference between Damask rose genotypes (Atashi, Bi-Khar, Chahar-Fasl, and Kashan) under 100 mM salinity on 14th day and 28th day after salinity treatment were illustrated in Fig. 5a,b which is confirmed that Atashi genotype have had better performance than others 28 days after salinity treatment.

## Discussion

Significant difference in Damask rose explants physiological traits such as leaf RWC and MSI was found among the four genotypes in this research (Fig. 1). RWC is undoubtedly one of the first plant traits influenced by salinity stress, because water stress occurs frequently when plants are exposed to high concentrations of salt in soil solution. Our findings revealed that salt stress affected on the water state of Damask rose genotypes, resulting in a decrease in RWC in saline conditions (Fig. 1a). Under control conditions, ʻAtashiʼ had the maximum leaf RWC (66.52%) (Fig. 1a). RWC decrease has been documented in several melon species, which is consistent with our findings^18^. Stevens et al.^19^ previously found that maintaining cell membrane integrity is important in salinity tolerance. MSI was reduced in this research when plants were exposed to salinity stress. Under saline conditions, ‘Atashi’ and ‘Bi-Khar’ had the most stable cell membranes (Fig. 1b). Several other researchers have confirmed these findings^20^, which stated that salinity increased cellular electrolyte leakage, so that selecting genotypes with superior leaf MSI may result in improved salinity tolerance in plants.

Salinity as well as drought stress, influenced on plant pigmentation as a decrease in the amount of photosynthetic pigments under salt stress is thought to be caused by delay in pigments biosynthesis or broken down quickly^21^. Reduced Chl *a* and Chl *b* levels, as well as total Chl content at higher NaCl concentrations, were measured in all Damask rose genotypes shoot (Fig. 2a–d). These findings are consistent with earlier research, which found that salt stress induced by NaCl, caused to decrease in total Chl concentration in *Phaseolus vulgaris*^22^. Salinity substantially reduced Chl content and photosynthetic efficacy of peppermint^23^. The enhanced activity of photosynthetic enzymes and the breakage of the pigment-protein complex could explain the decrease in Chl under salt stress^24^. According to Khan^25^, salt stress delayed the synthesis of photosynthetic pigments. The total carotenoid content of all Damask rose genotypes grown in vitro culture supplemented with 100 mM NaCl was reduced (Table 1). The findings were consistent with those found in *Cornus sericea*^26^ and melons^27^. Salt stress caused to β-carotene breakdown, which led to a lower carotenoid concentration^28^. Carotenoids are thylakoid membrane components that absorb and transmit light to Chl, protecting it from photooxidation^29^. As a result, the breakdown of carotenoids may also induce Chl degradation. The breakdown of photosynthetic pigments caused by salt is most likely due to higher toxic ions, increased chlorophyllase enzyme activity, and damage to the photosynthetic apparatus^30^. According to the recent study, a high amount of toxic ions because of the various salts in effluent caused to the loss of photosynthetic pigments in wheat, including Chl *a*, Chl *b*, and carotenoids^31^. Given that salinity changed photosynthetic pigment metabolism, it is evident that under abiotic stress, Chl content is tightly controlled^32^.

Salt stress can boost the actions of APX, CAT, and GR in *P. popularis* leaves^33^. The antioxidant enzymes (GPX, SOD, CAT, APX, PPO, and POD) in this study responded differently to salt treatment, exposure duration, and NaCl concentration (Table 2). Our findings indicated that antioxidant enzymes such as GPX, SOD, CAT, APX, and PPO in the leaf stayed active after prolonged activation (14th and 28th days), (Table 2). The activities of SOD, CAT, GPX, and APX in Damask rose explants decreased as well as increased in salinity duration (28th day) at sever salt stress (100 mM NaCl). These findings were in line with those reported for *Brassica juncea* L. in which antioxidant system activities increased after brief intervals of 30 min to 5 days of NaCl treatment^34^, and for maize seedlings treated for 21 days except for POD activity^35^. Numerous studies have shown that when plants were treated with NaCl, the activities of APX, SOD, CAT, and GR increased compared to control plants^36^. SOD and APX in mustard and maize^37^, and GPX, SOD, and CAT in UCB-1^38^. Under salinity, the transcript levels of the SOD, CAT, and APX genes stress significantly increased^39^. Furthermore, it has been demonstrated that GPX activity increased in corn under salinity stress^40^. When plants are subjected to salinity stress, ROS accumulation increased, resulting in an imbalance between ROS generation and scavenging, and cellular oxidative damage. Plants have been equipped with an effective scavenging ROS defense system that includes enzymatic (APX, SOD, CAT, GPX, POD) and non-enzymatic (carotenoids, flavonoids, and anthocyanin)^40^. Most likely, the levels of APX, GPX, CAT, APX, POD, and SOD in this research increased in order to reduce the amount of ROS generated under salinity stress. An increase in PPO activity is most likely an induced defensive response that delays senescence. The activity of PPO increased in response to salt stress with the highest activity in older leaves (Table 2). As an adaptive approach under stress, this could selectively promote cell death in these tissues, reducing further water loss and allowing limited nutrients to be partitioned to younger tissues. Previous research has shown that polyphenol production is influenced by abiotic variables such as ambient influences^41^. According to Radi et al.^42^, salinity-induced changes in plant metabolism could result in an accumulation in various phenolic compounds.

Under salt-induced stress, the total phenolic content increased in this research. Our findings revealed that as well as increase in NaCl level on the 14th and 28th days the total phenolic contents were also increased (Table 3). According to earlier study, increase in salt concentration significantly caused to increase in total phenolic contents level^43^. Furthermore, it has been demonstrated that salt stress can alter the phenolic molecules of plants, which are sensitive to salt^44^. The synthesis pathway of total phenolic content may be linked to its buildup under salinity stress. The phenylpropanoid pathway is responsible for the production of phenolic compounds in plants, and this pathway can be triggered in saline conditions^45^. Plants produced several hormones under salinity stress conditions, including methyl-jasmonic acid and jasmonic acid, which trigger phenylalanine ammonia lyase activity which is the primary enzyme in phenolic metabolism in plants results in the production of phenolic compounds^46^. In this study, flavonoid content was tested at various salt levels, and along with increase in NaCl concentration, flavonoid content increased on the 14th and 28th days in all Damask rose genotypes (Table 3). Gengmao et al.^47^ demonstrated that as well as increase in NaCl concentrations up to 150 mM, flavonoid levels increased in safflower plant. It was also stated that under abiotic stresses, plants boost flavonoid synthesis to protect themselves from unfavorable stress circumstances^48^. Plants use secondary metabolites in reaction to salinity stress to eliminate and cope to the harmful effects of salt stress. Plants use oxidative defense metabolism, compatible solutes, inorganic nutrients, hormonal control, and secondary compounds in reaction to salt stress^49^. Flavonoids are essential secondary metabolites that decrease the negative effects of osmotic stress, such as salinity^50^. Flavonoids accumulate in vacuoles, chloroplasts, and nuclei, and vacuole flavonoids interfere with hydrogen peroxide detoxification^51^. Salt stress effects on plant water status by lowering the osmotic potential of the soil solution^52^. As previously reported^53^, the total antioxidant capacity (TAC) system increased with increasing in salinity level. At moderate salinity, a small increase in antioxidant molecules and their activity implies a regulatory response. Significantly greater quantity and activity of these compounds, on the other hand, represent an apparent state of urgency to deal with oxidative stress under high salinity, which is also reflected in a significant growth decrease (Table 3).

Lower protein loss in of ‘Atashi’ may be attributable to its increased tolerance to NaCl stress conditions. Furthermore, these findings showed that extending the time exposure of salinity, reduced the amount of proteins, particularly in ‘Chahar-Fasl’ (Table 4). Plants have a dual reactivity to protein patterns when exposed to salt. Salt stress decreases overall protein concentration while simultaneously initiating the synthesis of other specialized proteins required for salt tolerance via ABA activation^54^. The increased activity of protease may be responsible for the breakdown of protein content in a saline soil. Degraded proteins may provide a storage form of nitrogen, which is important in osmotic regulation under stress^55^.

Proline content of in vitro cultivated Damask rose genotypes increased dramatically with increasing in salinity and time (Table 4). Our findings are consistent with previous studies of figure^56^. Proline performs critical functions such as osmoregulation and lowering the negative effects of ROS during salinity stress. As a result, more proline accumulation in plants can result in greater saline tolerance^43^. Under extreme salt stress, ‘Atashi’ had the greatest proline concentration in the current research (Table 4).

Based on what we found, increase in the level of NaCl causes H_2_O_2_ levels to increase in all genotypes on days 14 and 28 after salinity treatment. Several studies have shown that when plants are under stress from salt, the amount of H_2_O_2_ in their tissues increases more^36^. Under stress conditions, plants produce ROS, and an excessive buildup of ROS in response to salt stress can cause oxidative stress and severely damage natural metabolism via membrane lipid peroxidation^57^. In this study, salinity increased the MDA level of treated genotypes significantly on 14th and 28th days (Table 4). Increased lipid peroxidation is a frequent sign of salt stress, which is caused by an increase in ROS production. It has been said that increased ROS formation may harm cellular organelles and components such as membrane lipids, whereas genotypes with lower MDA concentration are more resistant to salty conditions^58^. In this aspect, ‘Bi-Khar’ has the lowest MDA content.

## Conclusion

The change in weather conditions due to global warming and increase in drought has led to an increase in soil salinity. Therefore, it is so important to measure the threshold of tolerance of different Damask roses to salt stress regarding to the way of defense mechanism. According to the results, it is clear that despite the severe stress applied to the Damask rose genotypes, the mechanisms involved in stress tolerance have worked well in these samples and the osmotic and antioxidant protection mechanisms against the death and deterioration of the explants. Although PPO was not activated under more concentrations of NaCl, POD and APX were the most common enzymes which kept their activation in 100 mM NaCl until 28th day after salinity treatment. Also TPC and TFC was in higher content until 28th day in tolerant Damask rose genotype, while other antioxidative enzymes as well as other osmolytes were up-regulated more at 14th day after salinity treatment. Based on the results we have selected the Atashi > Bi-Khar > Kashan > Chahar-Fasl genotypes as the most tolerant Damask rose under salt stress, respectively. So, it was concluded that Atashi genotype under 100 mM NaCl, was the most favorable and tolerant genotype with the highest optimal level of antioxidant activity and the accumulation of compatible osmolytes, as well as the high concentration of total soluble protein compared to the other genotypes. In conclusion, in vitro selection methods can be used effectively for salt tolerance screening of Damask rose genotypes, although the same experiment should be done in the field to verify the in vitro experimental results.

## Materials and methods

### Plant materials and culture medium

One-year branches with active growth of four genotypes of *R. damascena* including Chahar-fasal, Kashan, Atashi, and Bi-khar were taken from a research farm in Pakdasht, Iran. The plant material and shoots for wild collections were obtained under the supervision and permission of the Maragheh University guidelines and according to the national guidelines and all authors complied with all the local and national guidelines. Damask rose cuttings were washed 20 min under running tap-water, then they were surface-sterilized in 30% (v/v) NaClO for 20 min. The branches recut into the one node shoot explants (1.5–2 cm), and placed in the MS free hormone media^59^, for the initial establishment which solidified with 7.5 g of agar. The pH value was adjusted to 5.7 before autoclaving at 121 °C for 20 min. Five Shoot nodes were cultured, in 150 mL culture vessels containing 25 mL of establishment medium as one replication, then kept at 25 ± 2 °C and 16 h-photoperiod (light intensity, 8.85 W/m^2^) and 60–70% RH. Following explants were screened every day for fungal or bacterial contamination then any contaminated vessels were removed from the experiment collection. Shoots were repeatedly sub-cultured three times at a constant three-week subculture interval.

### Proliferation medium and growth of culture

The basic medium for proliferative media was the same as the MS establishment medium^59^, plus 130 mg of iron sequester and 332 mg of CaCl_2_, as well as 0.36 mg L^−1^ benzyl adenine (BA) and 0.03 mg L^−1^ Indole 3-butyric acid (IBA). The pH was then adjusted to 5.7 and sterilized in an autoclave. After sterilization and transfer to the flow-box, 25 mL of medium was added to each culture vessels to be used for subculture. Finally, five explants were placed in 150 mL culture vessels containing the proliferation medium as one replication and transferred to the growth chamber to be propagated and then the mentioned propagated shoots have been used.

### Preparing the treatment medium including NaCl to induced salinity

NaCl was added to the culture medium at five levels (0, 25, 50, 75 and 100 mM) to simulate salt stress. Shoot explants with only four young leaves were placed in culture medium, and then five explants were placed in each culture vessel and transferred to the growth chamber and after 14 and 28 days, the explants were collected and evaluated for physiological, and biochemical traits.

### Physiological traits

#### Relative water content (RWC)

Fresh leaf samples were weighted to determine fresh weight (FW), and then they were immersed in dH_2_O at 4 °C for 24 h. After absorption of surface water, they were weighed again for turgid weight (TW). Leaf samples were then dried in the oven at 75 °C for 48 h and their DW was measured and RWC was calculated according to the Barrs and Weatherley^60^ protocol.

#### Membrane stability index (MSI)

Membrane stability of Damask rose explants were evaluated by 0.1 g of leaves. Then similar disks were detached of them. The samples were put in test tubes having 10 mL of dH_2_O in two sets. Then the electrical conductivity of the samples were recorded after incubation at 40 °C for 30 min (C1) and 100 °C for 15 min (C2) by means of EC meters (Jenway model, UK) and the protocol of Almeselmani et al.^61^.

#### Chlorophyll *a*, *b*, total chlorophyll and carotenoids (CARs)

About 0.5 g of finely mixed of leaves were extracted using 10 mL of 80% acetone. After 24 h incubation in dark place chlorophyll was extracted from completely blanched leaves. Then the amount of absorbance of extracted samples was recorded at 649, 665, and 470 nm by a spectrophotometer (Shimadzu UV-2550, Kyoto, Japan). For evaluating the concentration of chlorophyll, a standard method similar to Arnon^62^, was applied.

### Biochemical traits

#### Antioxidative enzymes

Fresh leaves (1 g) were homogenized in 5 mL of 50 mM K–phosphate buffer (pH 7.0), brought to 5 mM Na–ascorbate and 0.2 mM EDTA by the addition of concentrated stocks. The homogenate extract was centrifuged at 10,000 rpm for 15 min at 4 °C. The supernatant was used for assessment of enzyme activities. Guaiacol Peroxidase (GPX) was evaluated by monitoring the increase in absorbance at 470 nm (ε = 26.6 mM^ −1^ cm ^−1^) during polymerization of guaiacol. One unit of activity was defined as the amount of enzyme producing 1 µmol of tetraguaiacol per min at 25 °C. Superoxide dismutase (SOD) was assayed according to the protocol of Beauchamp and Fridovich^63^, which is based on prevention of the photochemical reduction of nitro blue tetrazolium. The reactants were then placed under a 20-W fluorescent lamp for 15 min and the samples in the tubes were covered with a black cloth. At the end of the reaction, the absorbance at 560 nm was determined. The activity of catalase (CAT) was measured, as previously established by Li et al.^64^. The Damask rose explant leaves (0.5 g) were grounded in liquid nitrogen and extracted with the following described method: 100 mM potassium phosphate buffer (pH 7.8) including 0.1 mM EDTA, 1% (w/v) PVP and 0.1% (v/v) Triton × 100. The extraction was centrifuged at 10,000 rpm for 15 min at 4 °C. The supernatants were collected and applied for evaluating the enzyme activity. Ascorbate peroxidase (APX) activity was done according to the Yoshimura et al.^65^ protocol. The reaction solution involved phosphate buffer (250 μL), 1 mM ascorbate (250 μL), 0.4 μM EDTA (250 μL), 190 μL ddH_2_O, 10 mM trans oxide (10 μL) and 50 μL supernatant. The absorption at 290 nm for 1 min determined the enzyme activity. The activity of polyphenol oxidase (PPO) was measured by Nicoli et al.^66^ protocol. The enzymatic solution was extracted the same as peroxidase. In this case, a reaction complex was prepared of 100 μL of enzymatic extraction, 2.5 mL of potassium phosphate buffer (pH 6.8), 200 μl of 0.02-M pyrogallol as the enzyme precursor and was recorded at 420 nm with a spectrophotometer (Shimadzu, Japan).

#### Total phenol content (TPC)

Using the Folin-Ciocalteu reagent, the total phenolic concentration of Damask explants was determined as Singleton et al.^67^ method. The plant extract was combined with 0.5 cc of the Folin-Ciocalteu reagent, and the mixture was then held at 25 °C for 8 min. After 8 min, 2 mL of sodium carbonate solution (7.5% concentration) was added to this solution, and its volume was then increased with water to 8 mL. After two hours, the total phenolic concentration was determined using a 725 nm wavelength. A calibration curve was created using gallic acid as the reference. The amount of mg gallic acid equivalents per gram of sample was used to indicate the overall phenolic content.

#### Total flavonoid content (TFC)

Using quercetin as a reference standard, the total flavonoid was measured using the aluminum chloride technique^68^. To accomplish this, 1 mL of quercetin extract or standard solution (20, 40, 60, 80, and 100 μg mL^−1^) was added to a 10 mL volumetric flask containing 4 mL of purified water. After 5 min, 0.30 mL of 5% NaNO_2_ was added to the flask, followed by 0.3 ml of 10% AlCl_3_. After 5 min, 2 mL of 1 M NaOH was added, and the amount was increased to 10 mL with dH_2_O. The overall flavonoid concentration was determined at 510 nm. Total flavonoid amounts were recorded as mg quercetin equivalent (mg QE 100 g^−1^ FW).

#### Total antioxidant activity (TAA)

The 1,1-diphenyl-2-picrylhydrazyl (DPPH) technique was used to calculate TAA. 1 g of the fresh leaf samples was centrifuged at 21,913 g for 15 min after being separated with 2 mL of methanol at a concentration of 80%. After that, 100 µL of the extract was combined with 180 µL of DPPH (0.1 M), the mixture was left to sit in the dark for 30 min, and then a UV spectrophotometer was used to read the absorbance at 517 nm. The TAA was determined by applying the following equation^69^:

$$\mathrm{\%TAA}=\frac{\mathrm{OD\, control }-\mathrm{ OD\, sample}}{\mathrm{OD\, control}}\times 100$$

#### Protein

Protein content was assayed following the Bradford method^70^, which was calibrated with the standard bovine serum albumin curve. So, 100 mg of the treated explants were placed in a test tube with 2 mL of 50 mM potassium phosphate buffer at pH 7.0. The solution was centrifuged at 7000–12,000 rpm. Then, the supernatant was taken and centrifuged at 3000 rpm for 15 min at 4 °C. The samples were prepared with 1:100 dilution ratios and read at 595 nm.

#### Proline

Proline content was evaluated by homogenizing 0.2 g of leaf fresh weight in 2 mL of 3% aqueous sulfosalicylic acid and then centrifuged at 10,000 rpm for 30 min. Then, the pellet was washed with 3% aqueous sulfosalicylic acid twice. The supernatant was pooled, and the amount of proline was evaluated using ninhydrin reagent and toluene extraction^71^.

#### Hydrogen peroxide (H_2_O_2_)

Explants H_2_O_2_ content was determined according to the Liu et al.^72^ protocol. So, 0.5 g of leaf tissue was grounded with potassium phosphate buffer (KPB) (pH 6.8), and then centrifuged at 7000 rpm for 25 min at 4 °C. A 100-µL aliquot of the supernatant was added to 1 mL of Xylenol solution. The solution was then completely mixed and left to rest for 30 min and the extract absorption was read by a spectrophotometer (Shimadzu, Japan) at 560 nm.

#### Malondialdehyde (MDA)

MDA content was determined as 2-thiobarbituric acid (TBA) reactive metabolites^73^. So, 1.5 mL of the leaf extracts were homogenized in 2.5 mL of 5% TBA made in 5% Trichloroacetic acid. The mixture was warmed at 95 °C for 15 min and then cooled on ice quickly and centrifuged at 5000 rpm for 10 min. Then the absorbance of supernatant was read at 532 nm. MDA content was calculated as in the below equation:

$$\mathrm{MDA}=\frac{(\mathrm{A}532 -\mathrm{ A}600)}{155 }\times 1000$$

### Statistical analysis

The factorial experiment was carried out based on a completely randomized design with 3 replications. Data were statistically analyzed by MSTAT-C ver 2.1 software and means were separated using the Fischer’s protected least significant difference (LSD) test at p = 0.05. Pearson correlation and cluster dendrogram heat maps were performed in R foundation for sta-tistical computing (version 4.1.2), Iran (2021). URL https://cran.um.ac.ir/, accessed on3 June 2022.

### Plant permission statement

We obtained permission for collection of plant material used in the study.

## Data Availability

Correspondence and requests for the datasets generated and/or analyzed during the current study and materials should be addressed to H.S.H.
